# Surgical Resection of Intraocular Tumors (Partial Transscleral Sclerouvectomy Combined With Mircoinvasive Vitrectomy and Reconstruction of the Eyeball) in Asian Patients: Twenty-Five Years Results

**DOI:** 10.3389/fonc.2022.768635

**Published:** 2022-03-15

**Authors:** Nan Zhou, Ping Wang, Xiaolin Xu, Yueming Liu, Wenbin Wei

**Affiliations:** Beijing Tongren Eye Center, Beijing Key Laboratory of Intraocular Tumor Diagnosis and Treatment, Medical Artificial Intelligence Research and Verification Laboratory of the Ministry of Industry and Information Technology, Beijing Tongren Hospital, Capital Medical University, Beijing, China

**Keywords:** surgical resection procedure, intraocular tumors, globe salvage, useful vision, control of tumors

## Abstract

**Objective:**

To describe the outcome of intraocular tumor resection by partial transscleral sclerouvectomy (PTSU) combined with micro-invasive vitrectomy and reconstruction of the eyeball (MVRE) in Asian patients.

**Design, Methods and Participants:**

This retrospective, interventional cohort study included 366 patients who underwent PTSU combined with MVRE for intraocular tumors both in adult and pediatric age groups. The medical records of these patients were reviewed for clinical, operative, and histopathological features.

**Main Outcome Measures:**

Globe salvage, best corrected visual acuity (BCVA), surgical side effects, tumor control, and tumor-related metastasis and death.

**Results:**

The mean follow-up duration was 87 months (median, 66; range, 1-303 months). Among the 366 patients, the mean age was 8.5 years (median, 7; range, 1-19 years) in the 37 pediatric patients, and was 43 years (median, 42; range, 20-51) in 329 adult patients. The tumor mainly involved the ciliary body (n=136; 37.2%) and choroid (n=86; 23.5%). The common pathologic diagnosis of the 366 patients was as follows. In the pediatric age group, histopathologic examination revealed positive tumor margins in 37 patients mainly including ciliary body medulloepithelioma (8/37), ciliary body melanocytoma (13/37) and uveal melanoma (5/37). In the adult group, the pathological diagnosis mainly included melanoma (195/329), RPE adenoma (21/329), amelanotic melanoma (13/329), ciliary body adenoma of nonpigmented epithelium (19/329), schwannoma/neurilemmoma (11/329), melanocytoma (24/329), and leiomyoma (9/329). The globe salvage rate was 81.1% in the pediatric age groups (<20 years), and 93.6% in the adult group (≥20 years), respectively. Of the 338 salvaged eyes, final BCVA was 20/20 to 20/40 in 16 (4.7%), 20/40 to 20/80 in 58 (17.2%), 20/80 to 20/200 in 160 (47.3%), and ≤ 20/200 in 104 (30.8%). Early side effects included corneal edema in 28 (7.7%) patients, hyphema in 46 (12.6%), and vitreous hemorrhage in 76 (21%) patients. Postoperative side effects included proliferative vitreoretinopathy (PVR) in 67 (18.3%), late cataract in 42 (11.5%), and glaucoma in 18 (5%) patients. Local tumor recurrence was detected in 20 patients (5.5%) at a mean interval of 23.6 months, including melanoma (n=19) and medulloepithelioma (n=1). Enucleation was necessary in 28 (7.7%) cases owing to recurrence in 15 (53.6%), eye prophylaxis with high-grade malignancy in 5 (17.8%), and blind painful eye in 8 (28.6%) cases. Kaplan-Meier estimated for 5, 10-year metastasis rate and metastasis-related death rate (95%CI) in 213 UM patients were 3.2% (1.4%-7.0%), 6.9% (3.8%-12.3%); and 3.5% (1.6%-7.6%), 7.6% (4.2%-13.5%), respectively.

**Conclusions:**

As a surgically challenging procedure, PTSU combined with MVRE offers several theoretical advantages over enucleation and radiotherapy. It can achieve control of most intraocular tumors, preserve useful vision, and maintain a cosmetically normal eye.

## Highlights

Intraocular tumors are a rare condition, which not only spare vision but also endanger life. Choosing an appropriate management technique for controlling intraocular tumors, saving the eyeball, and preserving useful vision is important for improving the quality of life of patients.

## Introduction

Intraocular tumors are a rare condition in the population, which not only can cause loss of vision, but also endanger life. The incidence and prevalence of intraocular tumors occurring in pediatric patients and adults are different, and the same type of neoplasm maybe has different clinicopathological features in pediatric patients and adults. There are several benign tumors, malignant tumors, and simulating lesions that can occur in the eyes of the pediatrics or adults, including retinoblastoma, uveal melanoma (UM), hemangioma, medulloepithelioma, nevus, iris and ciliary body melanocytoma, and others. Treatment strategies for the management of these lesions included observation for benign, nonprogressive lesions and intervention for malignant or progressively enlarging tumors. Depending on the size, location and type of the tumor, interventional methods included cryotherapy, thermotherapy, surgical excision, plaque brachytherapy, laser photocoagulation, radiotherapy, chemotherapy, and enucleation (1). The treatment purpose for intraocular tumors is to control the tumor, prolong life, and preserve the eyeball and even useful vision. Surgical resection can be performed as a primary treatment or as a salvage procedure after another form of therapy.

Resection (surgical excision) is a technically challenging method in which a “trap-window” is created in the eye to allow entry and removal of a mass, followed by subsequent globe repair. It is designed to preserve useful vision and maintain a cosmetically normal eye. This technique is mainly used for ciliary body, choroidal and pigment epithelial tumors, and rare retinal tumors. Resection is classified based on the tissue involved as iridectomy, iridocyclectomy, iridogoniocycletomy, iridocyclochoroidectomy, iridogoniocyclochoroidectomy, partial lamellar sclerouvectomy (PLSU) (1–5), or local resection *via* 23- to 25-gauge micro-invasive vitrectomy for the excision of intraocular tumors, which we have termed as partial transscleral sclerouvectomy (PTSU) combined with micro-invasive vitrectomy and reconstruction of the eyeball (MVRE). Previous studies have termed surgical excision “en bloc” or “eye wall” resection (6–8). There are few previous studies that have evaluated the success of surgical resection for UMs in Caucasian adults (2–11), while the published literature is lacking the outcomes of local resection of intraocular tumors in the Asian population. Herein, to further explore the potential benefits of this surgical procedure, we reported an analysis of the outcomes of PTSU combined with MVRE for intraocular tumors in the pediatric and adult groups in the Asian population.

## Methods

The clinical records of all patients who underwent PTSU combined with MVRE from Jun 6, 1995 to Oct 26, 2020 at the Beijing Tongren Hospital were analyzed. The study and data collection were compliant with the principles of the Declaration of Helsinki. The study was approved by the Institutional Review Board of Beijing Tongren Hospital, and written informed consent was obtained from all participants. Asian patients who underwent surgery were included in this study; those under the age of 20 years were included in the pediatric group and those over the age of 20 years were in the adult group. The operative procedure was performed by senior ophthalmologists (WB. W).

The collected clinical data included patient demographics, associated ocular and systemic disease, treatment history, ocular symptoms, best corrected visual acuity (BCVA), and intraocular pressure (IOP). The evaluated tumor characteristics included tumor location, tumor configuration (mushroom, dome, plateau, or lentiform), tumor size (largest basal diameter and thickness in millimeters [mm]), surface features, color, pigmented (including dark black, brown or mixed) or nonpigmented, exudative retinal detachment, vitreous hemorrhage, secondary effects on adjacent structures, and extraocular extension. Largest tumor basal diameter and tumor thickness were measured by standard ocular Color Doppler ultrasonography (CDU). Surgical findings including intraoperative and postoperative period were noted. A record of the histopathological features was listed. Long-term outcomes such as tumor control, BCVA, surgical side effects, and tumor-related metastasis and death were assessed.

### Patient Selection

The decision to perform PTSU combined with MVRE was dependent on several factors, including tumor location, tumor size, tumor type, secondary effects of tumor, BCVA, and patient preference (3, 4). Other options for treatment based on the tumor type included observation, thermotherapy, plaque brachytherapy, and enucleation. Although the selection was case-based, PTSU combined with MVRE was generally applied for tumors measuring<18 mm in diameter and tumors with no evidence of vitreous invasion (3) or extraocular extension. Tumor thickness was a less important factor for considering PTSU combined with MVRE. For benign lesions, surgical resection was attempted only if the BCVA was compromised, causing secondary glaucoma, or if there was a high risk of amblyopia owing to astigmatism. For malignant lesions, the advantages and disadvantages of alternative treatments (such as plaque brachytherapy, radiotherapy and enucleation) were discussed with the patient before proceeding with PTSU combined with MVRE.

### Operative Procedure

#### PTSU Combined With MVRE

Local resection was performed in all patients *via* partial transscleral sclerouvectomy (PTSU) combined with 23 to 25-gauge micro-invasive vitrectomy and reconstruction of the eyeball (MVRE). The key operative procedure has been described in previous studies (1–3). For ciliary body or choroidal tumors, a limbal peritomy was created centered on the tumor meridian and the adjacent rectus muscles were isolated and tagged with 4-0 silk sutures. If the tumor margin underlaid a rectus muscle, then muscle disinsertion was performed, tagging with double armed 5-0 vicryl sutures. The episcleral blood vessels or sentinel vessels were cauterized gently and bare sclera was exposed over the tumor. The tumor margins were identified by transillumination and marked on the sclera with a marking pen. A ladder-shaped scleral flap was then created with a 2-mm margin from the tumor edge. In most cases, a posteriorly hinged flap was created to facilitate good wound edge apposition during the closure; however, when the tumor was anterior and within the iris and/or ciliary body, an anterior hinged flap was created. Ocular decompression by pars plana micro-invasive vitrectomy facilitated local excision by reducing retinal bulging through the scleral window. If the ciliary body tumor was small, a three-port vitrectomy was deemed unnecessary. A single infusion cannula was placed to stabilize intraocular pressure and reform the globe. Vitrectomy can be performed before, during, and/or after scleral flap dissection. Bipolar cauterization was performed on the uvea around the tumor for hemostasis. The uvea was carefully incised for the entire circumference of the tumor and cautious separation of the uveal mass from the underlying tissue (retina and vitreous) was subsequently achieved. For choroidal tumors, when the whole tumor body was separated from the sclera, floating in the pool of perfluorocarbon liquid, then the tumor was extracted through the scleral flap. The mass was safely removed and placed on a cardboard in formalin. Vitreous loss is often encountered when the ciliary body is the main area of tumor involvement because the thin nonpigmented ciliary epithelium is often adherent to the resected specimen (4, 5). In these scenarios, a 23 to 25-gauge micro-invasive vitrectomy was considered and performed. The scleral flap was closed with interrupted 8-0 nylon sutures depending on the location and size of the defect. The sutures were placed approximately 1.5-2 mm apart. The globe was reformed with balanced salt solution (BSS), injected through the pars plana. The structure of the eyeball was restored. The previously detached rectus muscle was resutured to its insertion and conjunctiva was reapproximated and closed with 8-0 vicryl sutures.

For iris tumors, either a scleral flap was made as described above or a limbal incision was created. A 15° microsurgical knife was used to create a paracentesis port 90° away from the main incision and a viscoelastic material was injected into the anterior chamber. The main incision was created using a 15° knife and is enlarged using a 3.2 microsurgical knife or 11th blade. The iris tumor was removed en bloc using Vannas capsulotomy scissors avoiding the pupillary rim. If the pupillary margin was removed, pupilloplasty was performed using 10-0 Prolene sutures in some patients. The anterior chamber was refilled with BSS and the flap or incision was sutured using interrupted 8-0 or 10-0 nylon sutures.

### Statistical Analysis

Data collected on continuous scale, including age (years), largest tumor basal diameter, and tumor thickness (millimeters), were expressed as mean, median, minimum and maximum. Kaplan-Meier analysis was performed to estimate the cumulative probability of metastasis and death. Factors relevant to metastasis and metastasis related death of UM patients after performing PTSU with MVRE were evaluated by univariate and multivariable Cox regression analyses. Hazard ratios and 95% CIs were calculated for each risk factor. P-value < 0.05 was considered to be statistically significant different. All analyses were performed using Stata version 15.0 (StataCorp LLC, College Station, TX, USA).

## Results

### Clinical Characteristics of Patients

In all, 366 patients (Asian/Chinese) who underwent PTSU combine with MVRE from Jun 6, 1995 to Oct 26, 2020. The mean age was 8.5 years (median, 7; range, 1–19) in the 37 pediatric patients, and was 43 years (median, 42; range, 20–51) in the 329 adult patients, respectively (**Tables 1**, **2**). Clinical features of the tumor are listed in **Table 1**.

Table 1ASurgical Resection (Partial Transscleral Sclerouvectomy Combine with Mircoinvasive Vitrectomy and Reconstruction of the Eyeball) of Intraocular Tumors in 37 Children: Demographic and Clinical Features.

**Table:** 

Features	Patients n
	n (%), n=37
**Age** (yrs), mean (median; range)	8.5, (7, 1-19)
**Gender**	
Male	21 (56.8%)
Female	16 (43.2%)
**Race**	
Asian	37 (100%)
**Tumor location**	
Iris	2 (5.4%)
Ciliary body	24 (64.9%)
Iris+ciliary body	2 (5.4%)
Ciliary body+choroid	2 (5.4%)
Choroid	4 (10.8%)
Retina	1 (2.7%)
Iris+ciliary body+choroid	2 (5.4%)
**Tumor size** (mm), mean (median, range)	
Largest tumor basal diameter	11.2 (10.5; 3.9-20.1)
Tumor thickness	6.5 (6.7; 1.5-12.1)
**Histopathologic diagnosis**	
** Medulloepithelioma**	8 (21.6%)
** Melanocytoma**	16 (43.3%)
Iris	1 (6.3%)
Ciliary body	11 (68.7%)
Iris+ciliary body	2 (12.5%)
Iris+ciliary body+ choroid	2 (12.5%)
**Melanoma**	5 (13.5%)
Pigmented	4 (80%)
Ciliary body-mixed cell	1 (25%)
Choroid-epithelioid	1 (25%)
Choroid-spindle cell	1 (25%)
Choroid-mixed cell	1 (25%)
Non-pigmented	1 (20%)
Choroid-mixed cell	1 (100%)
**Schwannoma**	1 (2.7%)
**Leiomyoma**	2 (5.4%)
**Nevus**	2 (5.4%)
Ciliary body	1 (50%)
Choroid	1 (50%)
**Inflammatory granuloma**	2 (5.4%)
**Hemangioma**	1 (2.7%)

**Table 1B:** Surgical Resection (Partial Transscleral Sclerouvectomy Combine with Mircoinvasive Vitrectomy and Reconstruction of the Eyeball) of Intraocular Tumors in 329 Adult: Demographic and Clinical Features.

Feature	Patients
	n (%), n=329
**Age** (yrs), mean (median; range)	43 (42; 20-51)
**Gender**	
Male	159 (48.3%)
Female	170 (51.7%)
**Race**	
Asian	329 (100%)
**Tumor location**	
Conjunctiva	4 ( 1.2%)
Iris	27 (8.2%)
Ciliary body	111 (33.7%)
Iris+ciliary body	16 (4.9%)
Ciliary body+choroid	55 (16.6%)
Choroid	82 (24.9%)
Retina	33 (10.1%)
**Tumor size** (mm), mean (median, range)	
Largest tumor basal diameter	9.1 (8.8; 1.5-22.2)
Tumor thickness	6.4 (6.6; 0.3-16.4)
**Histopathologic diagnosis**	
** Melanoma**	**208 (63.2%)**
Pigmented	195 (93.7%)
Conjunctiva	2 (1.1%)
Iris-spindle	13 (6.7%)
Iris-mixed cell	6 (3.2%)
Iris+ciliary body-spindle	5 (2.6%)
Iris+ciliary body-mixed cell	3 (1.6%)
Ciliary body-mixed cell	40 (20.6%)
Ciliary body-epithelioid	6 (3.2%)
Ciliary body-spindle	7 (3.6%)
Choroid-epithelioid	9 (4.6%)
Choroid-spindle cell	20 (11.0%)
Choroid-balloon	2 (1.1%)
Choroid-mixed cell	35 (17.9%)
Ciliary body + choroid-mixed cell	17 (8.8%)
Ciliary body + choroid-epithelioid	2 (1.1%)
Ciliary body + choroid–spindle	28 (14.4%)
**Non-pigmented**	**13 (6.3%)**
Ciliary body-epithelioid (1), spindle (1),mixed cell (4)	6 (46.2%)
Choroid-mixed cell (1), epithelioid (1)	2 (15.4%)
Ciliary body + choroid- spindle (4), epithelioid (1)	5 (38.4%)
**Melanocytoma**	24 (7.3%)
Iris	2 (8.3%)
Ciliary body	12 (50%)
Iris+ciliary body	8 (33.3%)
Choroid	2 (8.3%)
**Schwannoma (neurilemmoma)**	**11 (3.3%)**
Ciliary body	5 (45.5%)
Choroid	5 (45.5%)
Ciliary body+Choroid	1 (9.1%)
**Leiomyoma**	**9 (2.7%)**
Ciliary body	6 (66.7%)
Choroid	3 (33.3%)
**Inflammatory granuloma**	3 (0.9%)
**Retinoblastoma**	5 (1.5%)
**Malignant medulloepithelioma**	1 (0.3%)
**Ciliary body adenoma of nonpigmented epithelium**	19 (5.8%)
**CPE adenoma**	5 (1.5%)
**RPE adenoma**	21 (6.3%)
Pigmented	16 (76.2%)
Non-pigmented	5 (23.8%)
**RPE adenocarcinoma (choroid)**	1 (0.3%)
**RPE hamartoma**	1 (0.3%)
**Nevus**	1 (0.3%)
**Retinal capillary hemangioma**	2 (0.6%)
**MALT lymphoma**	4 (1.2%)
**Solitary fibrous tumor**	1 (0.3%)
**Glioneuroma**	2 (0.6%)
**Ameloblastoma**	1 (0.3%)
**Glomangioma**	1 (0.3%)
**Inflammatory pseudotumor**	1 (0.3%)
**Allelocytoma**	1 (0.3%)
**Neurofibroma (choroid)**	1 (0.3%)
**Amyloidosis**	2 (0.6%)
**Plasmacytoma**	1 (0.3%)
**Others**	
Iris metastatic tumor	3 (0.9%)
Iris foreign body	1 (0.3%)

CPE, ciliary body pigmented epithelial adenoma.

**Table 2:** Surgical Resection (Partial Transscleral Sclerouvectomy Combine with Mircoinvasive Vitrectomy and Reconstruction of the Eyeball) of Intraocular Tumors in 366 Patients: Early and Late Surgical Side Effects.

Side effects	n (%)
**Early postoperative side effects (< 2 weeks)**	
Corneal edema	28 (7.7%)
Hyphema	46 (12.6%)
Descemet’s folds	19 (51%)
Vitreous hemorrhage	76 (21%)
Elevated IOP	46 (12.6%)
**Late posterative effects (> 2 weeks)**	
Hypotony	2 (0.5%)
Proliferative vitreoretinopathy	67(18.3%)
Cataract	42 (11.5%)
Glaucoma	18 (5%)
Vitreous hemorrhage (nonresolving)	2 (0.5%)
Recurrent retinal detachment	7 (2%)
Sympathetic ophthalmia	1 (0.3%)

IOP, intraocular pressure.

**Figure 1:**
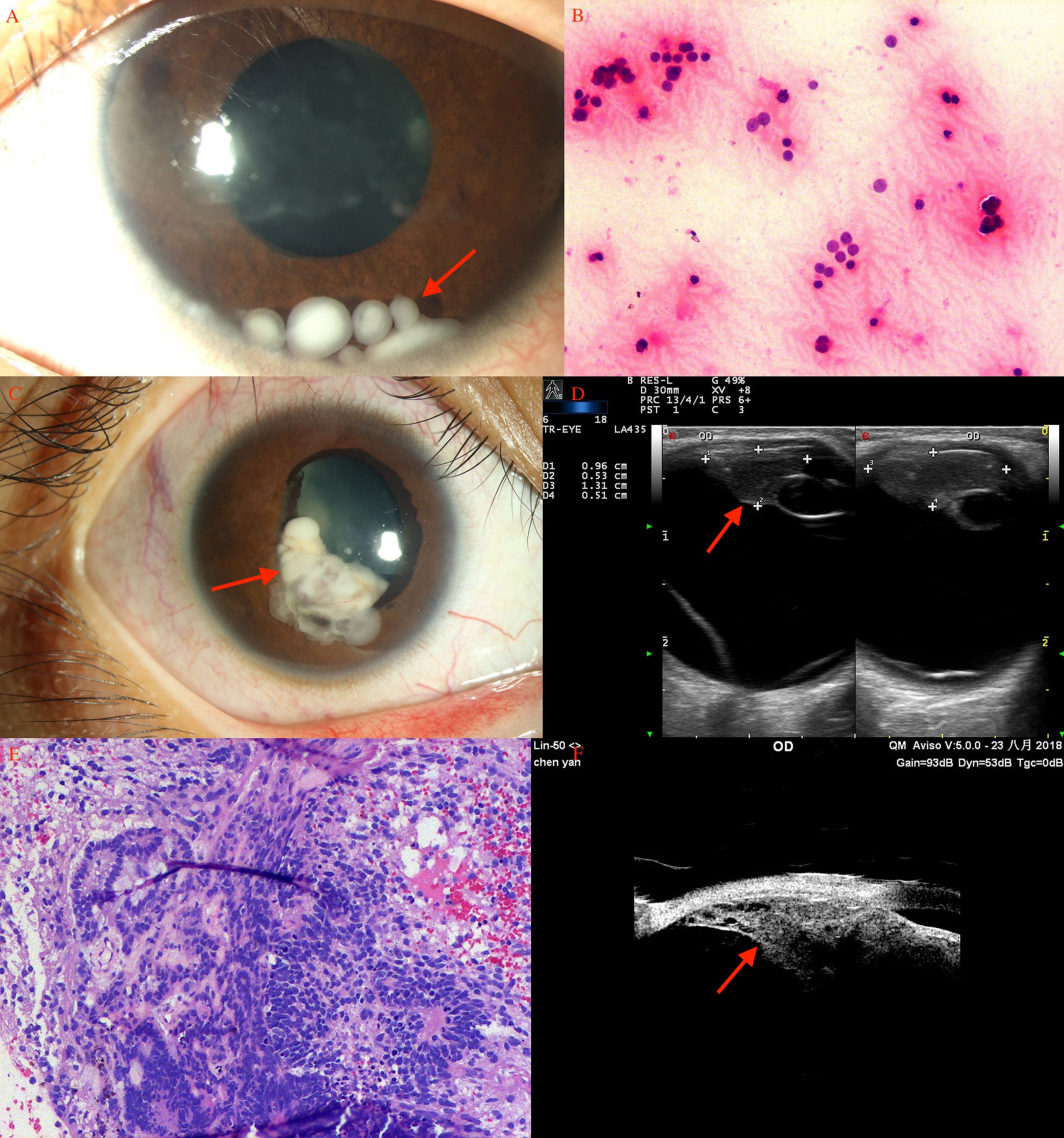
Ciliary body medulloepithelioma of a 5-year-old boy. **(A)** The pompom-like pseudohypopyon (arrow) in the anterior chamber (original magnifications × 16). (left eye) **(B)** The anterior-chamber fine-needle aspiration biopsy showed small, round blue tumor cells, with small bland nuclei and relatively abundant eosinophilic cytoplasm, arranged in nest bulk (hematoxylin-eosin [HE], original magnifications×200). **(C)** Medulloepithelioma of a 17-year-old girl. A markedly white tumor (arrow) in the pupil area with iris involvement (leukocoria), a prominent episcleral (sentinel) vessel, and rubeosis were noted (arrow). **(D)** CDI showed lesions (arrow) with low-to-moderate internal reflectivity and sharply demarcated borders; cystic components were seen. **(E)** Histopathology revealed ciliary body medulloepithelioma. **(F)** UBM of ciliary body tumor with iris invasion (iridociliary tumor, arrow) showed low-to-medium internal reflectivity and loss of the acute angle shape.

In the pediatric age group, BCVA at presentation was 20/20 to 20/40 in 2 (5%) eyes, 20/40 to 20/80 in 5 (13%), 20/80 to 20/200 in 6 (16%), and ≤ 20/200 in 24 (65%) eyes. The mean largest tumor basal diameter was 11.2 mm (median, 10.5; range, 3.9-20.1), and the mean thickness was 6.5 mm (median, 6.7; range, 1.5-12.1). The tumor was clinically pigmented in 22 (59.5%) and nonpigmented in 15 (40.5%) patients. The other associated findings at presentation included sentinel vessel in 30 (81%), corneal blood staining in 1 (3%), band-shaped corneal degeneration in 12 (32.4%), pseudohypopyon in 1 (3%) (**Figure 1A**), hyphema in 1 (3%), elevated IOP >21 mmHg in 2 (6%), rubeosis in 7 (19%), iris stromal seeding in 4 (11%), iris cyst in 2 (6%), dislocation of lens in 8 (21.6%), angle seeding in 1 (3%), cataract in 9 (24.3%), and feeder or drainer vessel in 1 (3%) patient.

In the adult group, BCVA at presentation was 20/20 to 20/40 in 33 (10%) of eyes, 20/40 to 20/80 in 76 (23%), 20/80 to 20/200 in 146 (44%), and ≤ 20/200 in 74 (23%) eyes. The mean tumor largest basal diameter was 9.1 mm (median, 8.8; range, 1.5–22.2), and the mean thickness was 6.4 mm (median, 6.6; range, 0.3–16.4). The tumor was clinically pigmented in 238 (72.3%) and nonpigmented in 91 (27.7%) patients. The other associated findings at presentation included sentinel vessel in 185 (56.2%), pseudohypopyon in 2 (0.6%), rubeosis in 2 (0.6%), iris cyst in 2 (0.6%), dislocation of lens in 66 (20.1%), angle seeding in 3 (0.9%), elevated IOP >21 mmHg in 10 (3%), cataract in 6 (1.8%), yellowish retinal exudation in 29 (8.8%), secondary exudative retinal detachment in 245 (74.8%), vitreous hemorrhage in 16 (4.9%), vitreous pigment dissemination in 8 (2.4%), exudative macular detachment in 56 (17%), surface wrinkling retinopathy in 22 (6.7%), and feeder or drainer vessel in 25 (7.6%) patients. The most relevant sign of ciliary body tumors is a prominent episcleral (sentinel) vessel.

### Intraoperative Course

Performed surgeries included iridectomy in 29 (8%), iridogoniocyclectomy in 18 (5%), iridogoniocyclochoroidectomy in 201 (55%), and cyclochoroidectomy in 84 (23%) patients. Limbal-based incision (with no flap) was used in 33 (9%) patients; a fornix-based flap was created in 293 (80%) patients. Muscle disinsertion was required in 6 (1.6%) patients to gain access to the surgical site. Standard three-port micro-invasive vitrectomy was performed in 337 (92%) patients for ensuring stabilized intraocular pressure and reattachment of the retina at resection (23 to 25-gauge). On completion of tumor removal, pupillary reconstruction was performed in 29 (8%) patients using Prolene sutures; repair with donated sclera was performed for sealing the wound in 6 (1.6%) patients. Intraoperative hyphema was noted in 40 (11%), vitreous hemorrhage in 70 (19%), and subretinal hemorrhage in 2 (0.5%) patients. No patient developed suprachoroidal expulsive hemorrhage.

### Pathology

**Figure 2:**
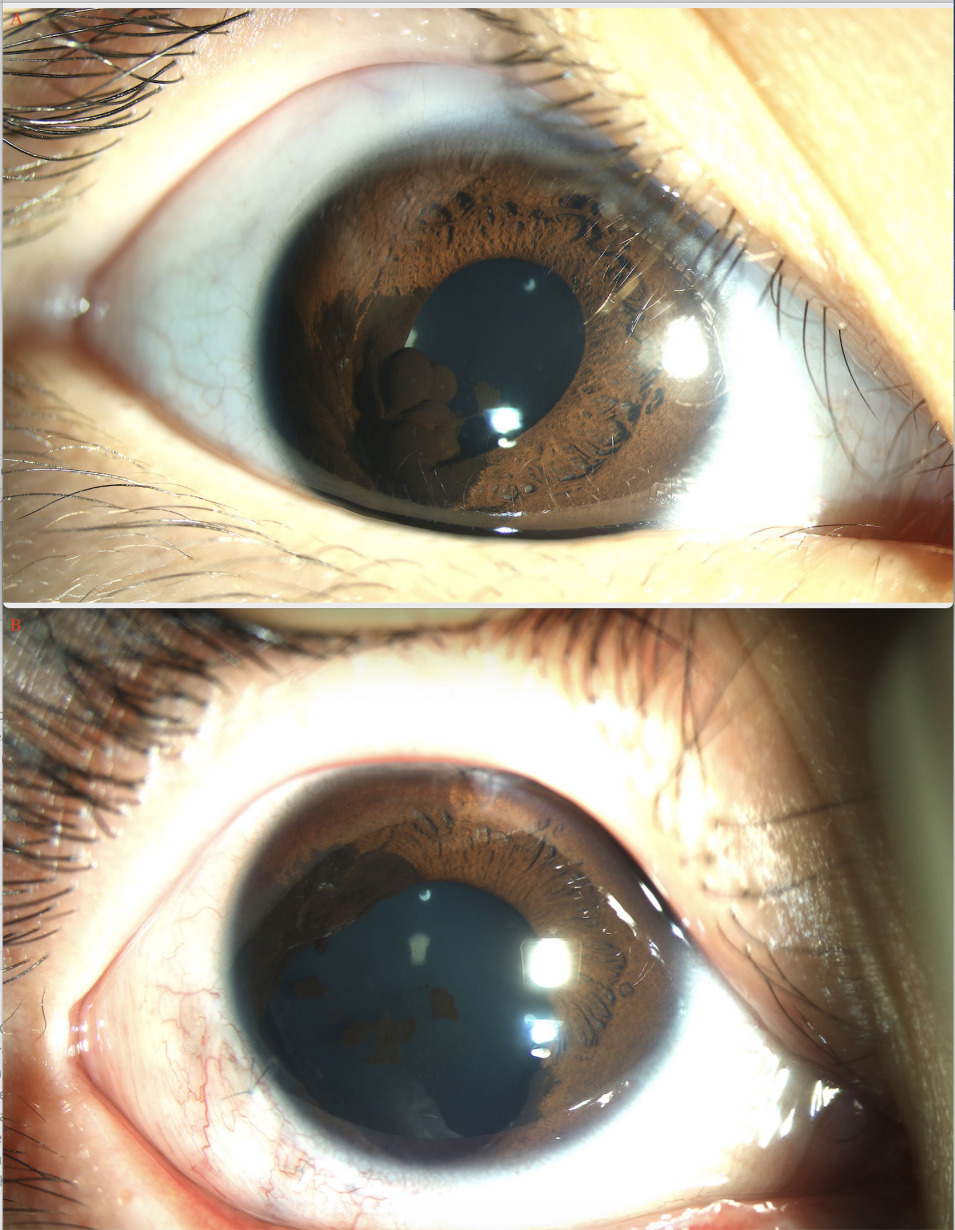
Iris melanocytoma (arrow) of a 7-year-old girl before **(A)** and after **(B)** iridectomy.

**Figure 3:**
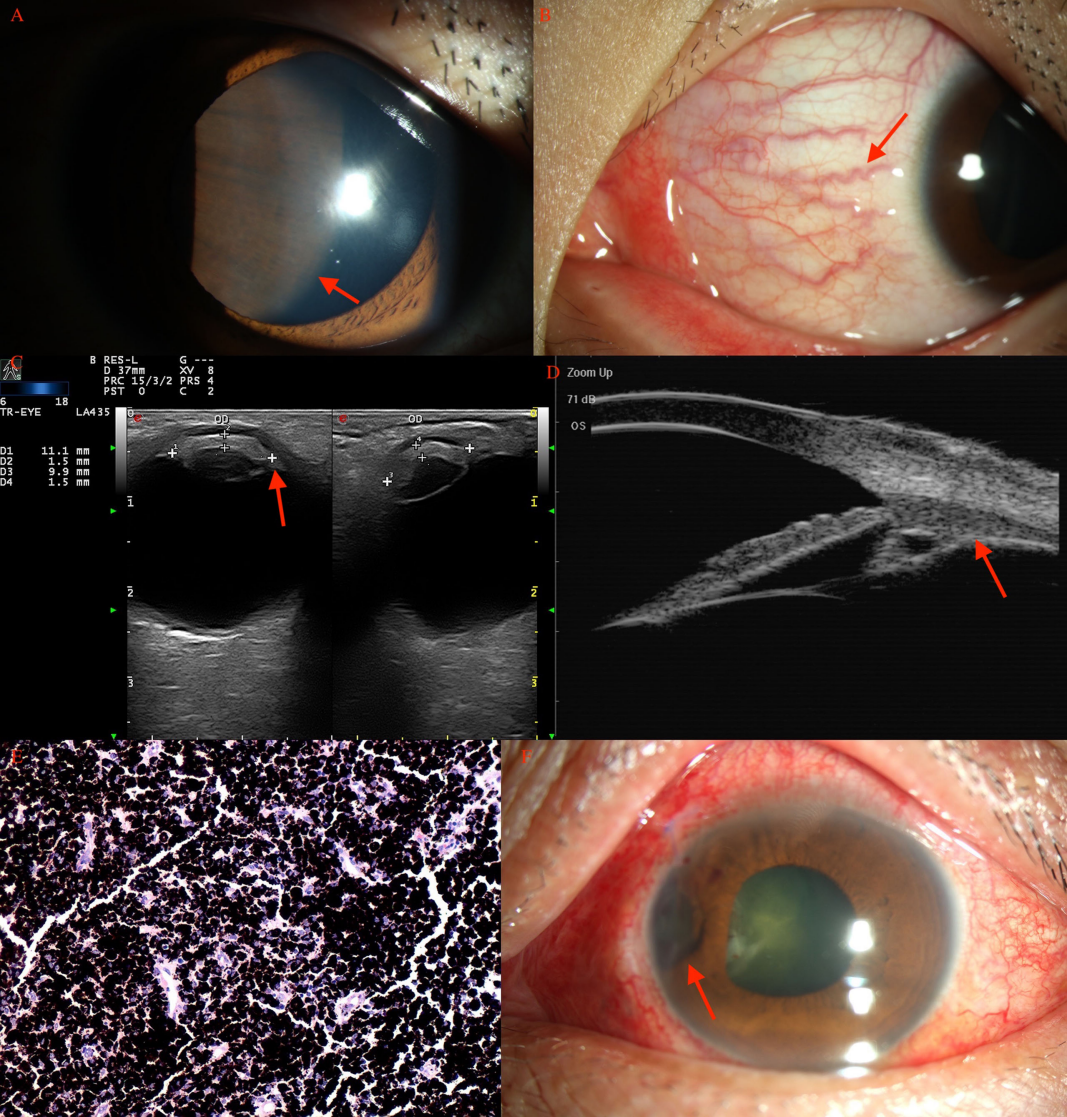
Ciliary body melanocytoma (**A**, arrow) of a 15-year-old boy, with prominent sentinel vessels (**B**, arrow). **(C)** CDI revealed lesions (arrow) with moderate internal reflectivity with irregular borders. **(D)** UBM showed a mass (arrow) pushing the normal iris posteriorly. **(E)** Histopathology revealed an amount of melanin in the tumor cells, with a small, round, normochromic, and regular nucleus. **(F)** After PTSU combined with MVRE.

The pathological diagnosis of the 366 patients is listed in **Tables 1**, **2**. In the pediatric age group, histopathological examination revealed positive tumor margins in 33 patients including those with medulloepithelioma (ciliary body in 8 patients; **Figures 1A–F**), melanocytoma (ciliary body in 13 patients, choroid in 2 patients, iris in 1 patient) (**Figures 2**, **3**), melanoma (ciliary body in 1 patient, choroid in 4 patients), leiomyoma (ciliary body in 2 patients), schwannoma (ciliary body in 1 patient), hemangioma (retina in 1 patient), and inflammatory granuloma (iris in 2 patients), nevus (ciliary body in 1 patient, choroid 1 patient).

**Figure 4:**
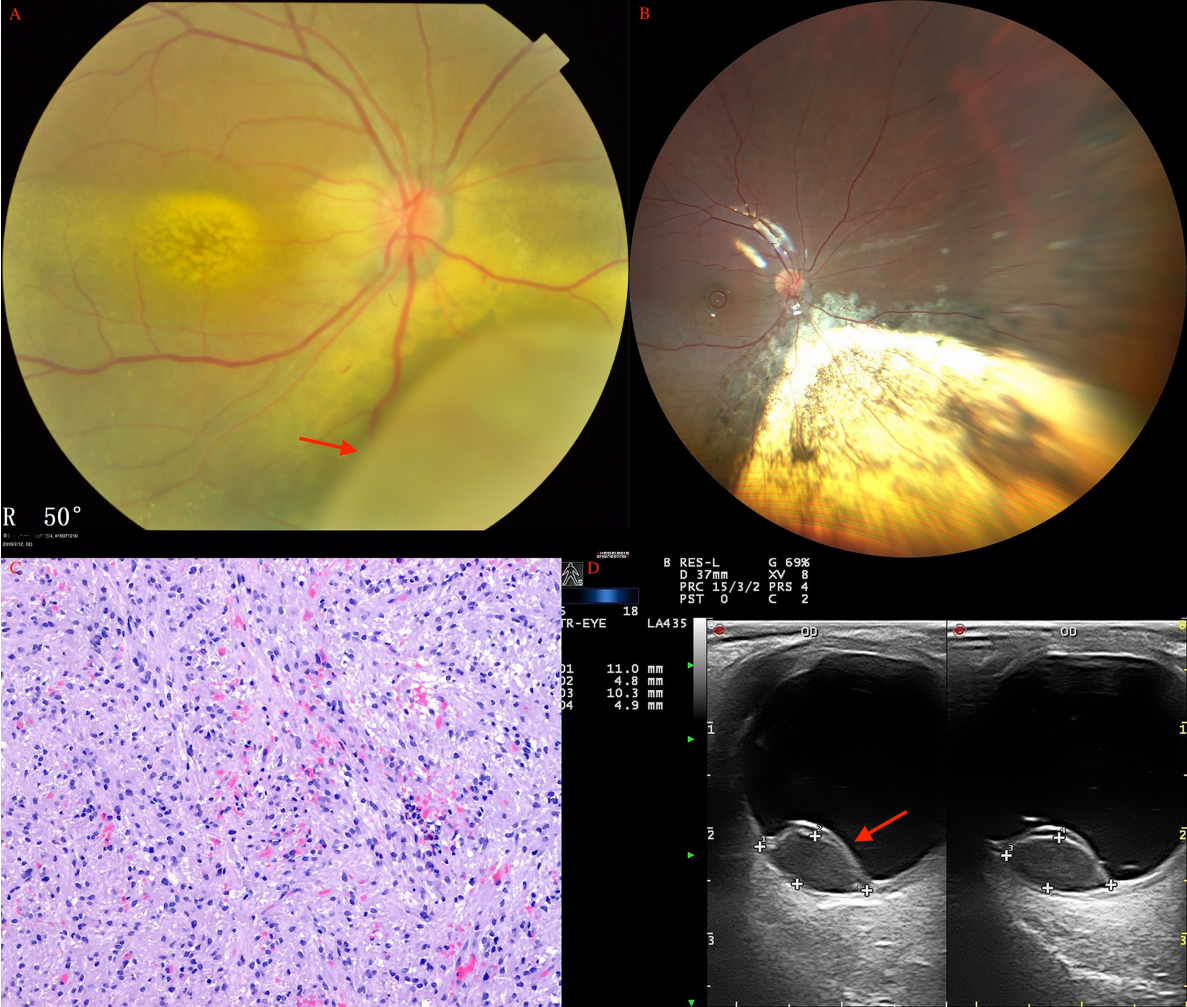
Choroidal leiomyoma (arrow) of a 27-year-old woman before **(A)** and after **(B)** PTSU combined with MVRE. **(C)** Photomicrograph of leiomyoma showing bundles of spindle cells with blunt-ended oval nuclei (HE, ×100). **(D)** CDI revealed a pedunculated mass (arrow) with inconsistent reflectivity of moderate intensity, and arterial blood signals in the tumor.

**Figure 5:**
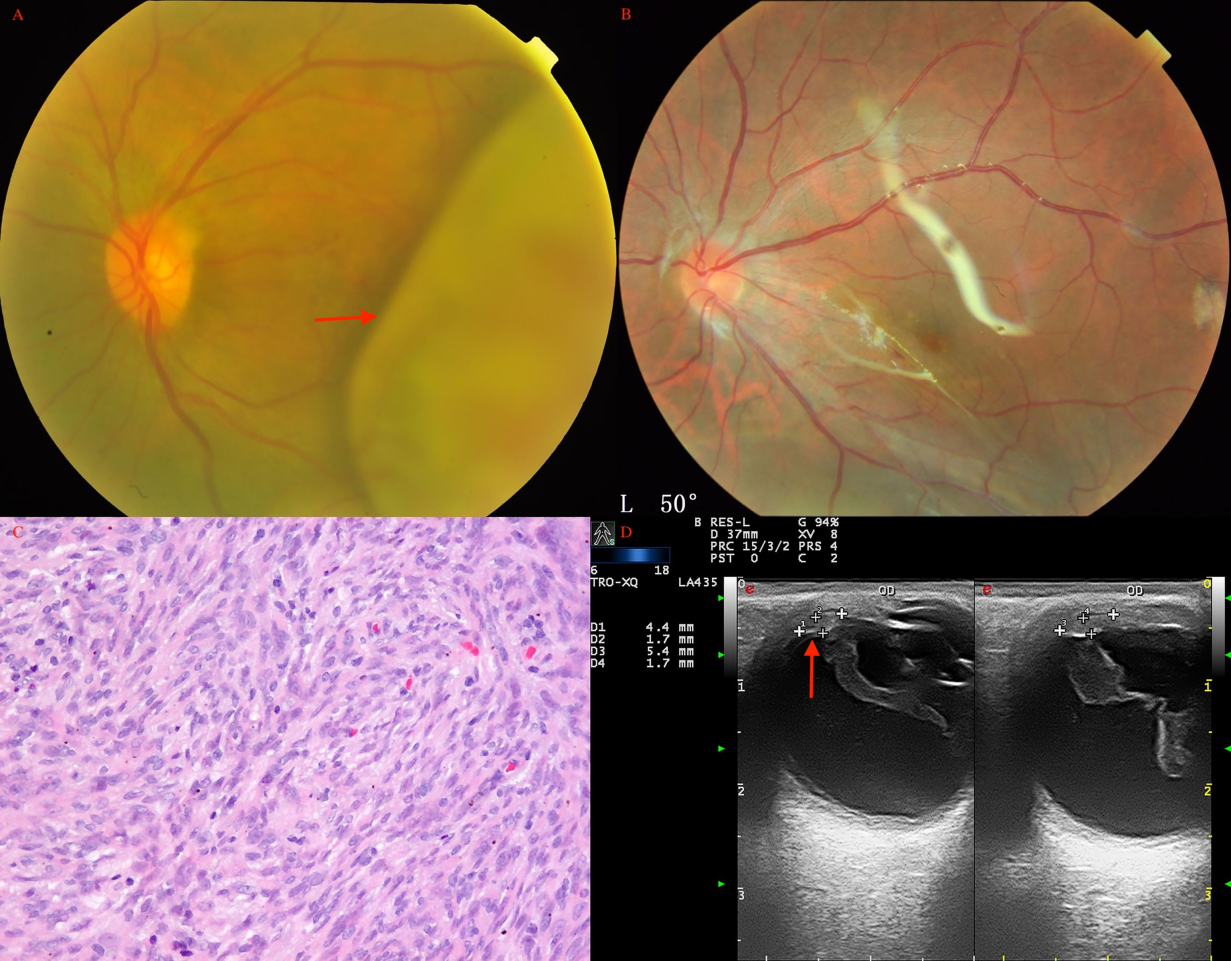
Choroidal schwannoma/neurilemmoma (arrow) in a 43-year-old man before **(A)** and after **(B)** PTSU combined with MVRE. **(C)** Histopathology revealed that the tumor consisted of spindle cells in a fascicular arrangement with long eosinophilic cytoplasmic processes and cells loosely arranged in a mucoid matrix (HE, × 200); **(D)** CDI showed two pedunculated masses (arrow) with inconsistent reflectivity of moderate intensity and no choroidal excavation, with arterial blood signals in the tumor.

In the adult group, histopathological examination revealed positive tumor margins in 321 patients including those with melanoma (conjunctiva in 2 patients, iris in 19 patients, ciliary body in 61 patients, choroid in 113 patients), amelanotic melanoma (ciliary body in 6 patients, choroid in 7 patients), malignant medulloepithelioma (ciliary body in 1 patient), RPE adenoma (21 patients), RPE adenocarcinoma (1 patient), ciliary body adenoma of nonpigmented epithelium (19 patients), leiomyoma (ciliary body in 6 patients, choroid in 3 patients; **Figure 4**), schwannoma/neurilemmoma (ciliary body in 6 patients, choroid in 5 patients; **Figure 5**), retinal hemangioma (2 patients), well-differentiated retinoblastoma (3 patients), melanocytoma (iris in 2 patients, ciliary body in 20 patients, choroid in 2 patients), amyloidosis (vitreous and retina in 2 patients), RPE hamartoma (1 patient), MALT-lymphoma (choroid in 1 patient), solitary fibrous tumor (choroid in 1 patient), ameloblastoma (choroid in 1 patient), glomangioma (ciliary body in 1 patient), glioneuroma (choroid in 1 patient, ciliary body and choroid in 1 patient), neurofibroma (choroid in 1 patient), inflammatory granuloma (iris in 2 patients, choroid in 1 patient), metastatic tumor (iris in 3 patients), allelocytoma (choroid in 1 patient), plasmacytoma (choroid in 1 patient), and inflammatory pseudotumor (choroid in 1 patient).

Among the 366 patients, 3 pediatric patients (0.8%) and 13 adult patients (3.6%) were incorrectly diagnosed preoperatively *via* clinical assessment. The preoperative incorrect diagnosis included 2 cases of medulloepithelioma (preoperatively diagnosed as retinoblastoma and hemangioma), 1 case of granuloma (preoperatively diagnosed as amelanotic melanoma), 13 cases of amelanotic melanoma (preoperatively diagnosed as neurilemmoma and atypical hemangioma), melanoma (preoperatively diagnosed as RPE adenoma), gliosis (preoperatively diagnosed as adenoma), melanocytoma (preoperatively diagnosed as melanoma), leiomyoma (preoperatively diagnosed as amelanotic adenoma), and a patient with nevus (preoperatively diagnosed as transscleral melanoma).

### Surgical Side Effects

Postoperative side effects are listed in **Table 2**. Early side effects included corneal edema in 28 (7.7%), hyphema in 46 (12.6%), and vitreous hemorrhage in 76 (21%) patients. Late side effects included proliferative vitreoretinopathy (PVR) in 67 (18.3%), late cataract in 42 (11.5%), glaucoma in 18 (5%), recurrent retinal detachment in 7 (2%), hypotony in 2 (0.5%), non-resolving vitreous hemorrhage in 2 (0.5%), and sympathetic ophthalmia in 1 (0.3%) patients. PVR occurred at mean 12 months after surgery. Postoperative cataract surgery was performed in 7 (19%) patients and scleral buckling was performed in 3 (11%) patients (2 cases of rhegmatogenous retinal detachment). The management of glaucoma included topical therapy for 10, trabeculectomy for 3, cyclophotocoagulation for 2, and glaucoma tube shunt for 3 patients.

### Outcomes

**Figure 6:**
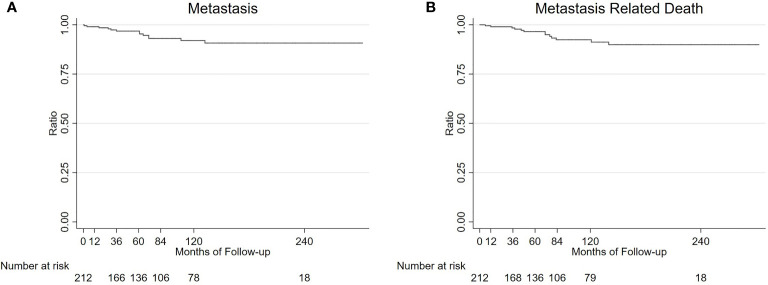
Survival analysis of 213 UM patients. Kaplan-Meier estimated that the 1, 3, 5, 7, and 10-year metastasis rates and metastasis related death rate (95% CI) in 213 UM patients were 0.5% (0.1%-3.3%), 3.2% (1.4%-7.0%), 3.2% (1.4%-7.0%), 6.9% (3.8%-12.3%), and 6.9% (3.8%-12.3%) **(A)**; and 1.0% (0.2%-3.4%), 1.6% (0.5%-4.8%), 3.5% (1.6%-7.6%), 7.6% (4.2%-13.5%), and 7.6% (4.2%-13.5%) **(B)**, respectively.

The mean follow-up duration was 87 months (median, 66; range, 1-303 months). Among the 366 patients, local tumor recurrence was detected in 20 (5.5%) patients at a mean interval of 23.6 months, including melanoma (n = 19) and medulloepithelioma (n = 1) (**Figure 6**). This was treated with I-125 plaque brachytherapy in 4 patients with melanoma and 1 patient with medulloepithelioma, and enucleation in 15 patients with melanoma. Overall, 28 (7.7%) patients underwent enucleation after a mean interval of 8.2 months (median 4.5; range, 1-26 months). The reason for enucleation was tumor recurrence in 15 (53.6%), prophylaxis for high-grade malignancy or tumors invading the optic nerve in 5 (17.9%), and blind painful eye caused by neovascular glaucoma (NVG) in 8 (28.5%) patients. Among the 338 salvaged eyes, the final BCVA was 20/20 to 20/40 in 16 (4.7%), 20/40 to 20/80 in 58 (17.2%), 20/80 to 20/200 in 160 (47.3%), and ≤ 20/200 in 104 (30.8%) patients (**Table 3**). There were no cases of orbital recurrence, metastasis or death at a mean of 97.0 months of follow-up.

Table 3ABCVA of 366 patients before and after surgery of PTSU and MVRE.

**Table:** 

BCVA	Pediatric age group		Adult group	
-	Before surgery (n=37)	After surgery (n=30)	Before surgery (n=329)	After surgery (n=308)
20/20~20/40	2 (5.4%)	1 (3.3%)	33 (10.0%)	15 (4.9%)
20/40~20/80	5 (13.5%)	0 (0%)	76 (23.1%)	58 (18.8%)
20/80~20/200	6 (16.2%)	8 (26.7%)	146 (44.4%)	152 (49.4%)
≤20/200	24 (64.9%)	21 (70%)	74 (22.5%)	83 (26.9%)
**Enucleation**	**n=7**		**n=21**

**Table 3B:** BCVA of 213 UM patients before and after surgery of PTSU and MVRE.

BCVA	Pediatric age group		Adult group	
-	Before surgery (n=5)	After surgery (n=2)	Before surgery (n=208)	After surgery (n=188)
20/20~20/40	0 (0%)	0 (0%)	39 (18.8%)	14 (7.4%)
20/40~20/80	0 (0%)	0 (0%)	39 (18.8%)	15 (8.0%)
20/80~20/200	0 (0%)	0 (0%)	84 (40.3%)	97 (51.6%)
≤20/200	2 (100%)	2 (100%)	46 (22.1%)	62 (33.0%)
**Enucleation**	**n=3**		**n=20**	

In this study, 4 patients (11%) underwent surgery at <3 years of age. The diagnoses in these patients were ciliary body medulloepithelioma (n = 3), and ciliary body melanocytoma (n = 1). After a mean follow-up of 40.0 months, no patient had a recurrence and the globe was salvaged in all patients. Infant eye surgeries are more challenging because of the increased pliability of the sclera causing an increased tendency to collapse during surgery. Hence, surgery was considered only if the lesion was obstructing the visual axis and inducing amblyopia in infants.

### Medulloepithelioma

In this study, medulloepithelioma was diagnosed in 8 (22%) patients in the pediatric age group (**Figures 1A–F**) and only 1 patient in the adult group. The lesions were primarily located in the ciliary body, and rubeosis (n=8, 89%) was a common manifestation combined with medulloepithelioma.

In the pediatric age group, the age group for medulloepithelioma ranged from 1 to 17 years, and it observed in 6 (75%) males and 2 (25%) females. Iridogoniocyclectomy was performed in all using a limbal-based flap in 2 (25%) and a fornix-based flap in 6 (75%) patients. Tumor recurrence was noted in 1 (11.1%) patient at 3 months. The recurrence was treated with I-125 plaque brachytherapy in 1 patient, but enucleation was ultimately required because of high-grade malignancy in pathological analysis. Medulloepithelioma was relatively common in children but usually was misdiagnosed during preoperative assessment. Medulloepithelioma in two patients was clinically diagnosed as retinoblastoma and hemangioma, and 2 patients with retinoblastoma were diagnosed to have medulloepithelioma preoperatively. After a mean follow-up duration of 22.5 months, no patient was noted to have documented metastasis.

Malignant medulloepithelioma that occurred in adults was extremely rare; only 1 patient with malignant medulloepithelioma was clinically diagnosed to have amelanotic melanoma or adenoma preoperatively.

### Melanocytoma

There were 16 (43%) pediatric patients and 24 (7.3%) adult patients with a histopathological diagnosis of melanocytoma in this study (**Figure 3**). Among pediatric patients, the age group for melanocytoma ranged from 3 to 9 years, and gender was male in 9 (56.3%) and female in 7 (43.8%). The lesions were primarily located in the ciliary body (n = 13; 81.2%) with anterior chamber angle involvement, choroid (n = 2; 12.5%), and iris (n = 1; 6.3%). Sentinel vessel (n = 16, 100%), corneal blood staining/corneal edema (n=13, 81.2%), and rubeosis (n=4, 25%) were common manifestations accompanying melanocytoma. Iridogoniocyclectomy was performed in all patients using a limbal-based flap in 6 (37.5%) and a fornix-based flap in 10 (62.5%). Melanocytoma was common in children and mainly located in the ciliary body with angle involvement; necrosis was noted in the tumor.

Among the adult patients, the age group for melanocytoma ranged from 20 to 56 years, and gender was male in 6 (25%) and female in 18 (75%). The lesions were primarily located in the ciliary body (n = 20; 83.4%), iris (n = 2; 8.3%), and choroid (n = 2; 8.3%). The elevated IOP>21 mmHg (n = 10; 41.7%), pigmented in iris (n = 22, 91.7%), optic atrophy (n = 9, 37.5%), and sentinel vessel (n = 18, 75%) were common manifestations accompanying tumors. Melanocytoma occurrence in adults was relatively common and was diagnosed clinically as melanoma preoperatively. Small ciliary body melanocytoma was commonly misdiagnosed as glaucoma and even a trabeculectomy surgery was performed (n = 6, 25%). Iridogoniocyclectomy was performed in all patients using a limbal-based flap in 8 (33%) and a fornix-based flap in 16 (66%). The intraocular pressure was normal after the surgery (n = 10).

### Melanoma

There were 213 patients with the histopathological diagnosis of melanoma in this study, 5 were pediatrics and 208 were adults. Among pediatric patients, the mean age was 6 years (median 5; range, 2–11 years). Among the 5 pediatric patients, 2 (40%) were male and 3 (60%) were female; 4 lesions were pigmented and 1 was non-pigmented. The tumors were primarily located in the ciliary body (n = 1; 20%), and choroid (n = 4; 80%). The pathological cell type was identified to be epithelioid in 1 (20%), spindle cell in 1 (20%), and mixed cell type in 3 (60%) patients. After a mean follow-up period of 148 months, 3 (60%) patients were found to have a recurrence and no patient had documented metastasis. Two cases of the recurrences were treated with enucleation and the third was treated with I-125 plaque brachytherapy.

Among adults, the mean age was 43.8 years (median, 44; range, 21–70 years). Among the 208 patients, 111 (53.4%) were male and 97 (46.6%) were female. The tumors were 13 in nonpigmented and pigmented in 195 patients. The tumors were primarily located in the ciliary body (n = 67; 32.2%), and choroid (n = 122; 58.7%). The pathological cell type was epithelioid in 20 (9.5%), spindle cell in 78 (37.5%), and mixed cell type in 106 (51.0%) patients. After a mean follow-up duration of 24.1 months, 16 (7.7%) patients were noted to have a recurrence with 13 patients had developing hepatic metastasis, and 3 patients along with orbit extension; 13 patients with recurrences were treated with enucleation and 3 patients were treated with I-125 plaque brachytherapy.

Kaplan-Meier analysis estimated that the 1, 3, 5, 7, and 10-year metastasis rates and metastasis related death rates (95% CI) in 213 UM patients were 0.5% (0.1%-3.3%), 3.2% (1.4%-7.0%), 3.2% (1.4%-7.0%), 6.9% (3.8%-12.3%), 6.9% (3.8%-12.3%); and 1.0% (0.2%-3.4%), 1.6% (0.5%-4.8%), 3.5% (1.6%-7.6%), 7.6% (4.2%-13.5%),7.6% (4.2%-13.5%) (**Figure 6**), respectively. Multivariable analysis showed no significant factors having different relative risks for metastasis and metastasis related death, including older age, sex, tumor base, tumor thickness, color and pathological type (**Supplementary Tables 1**, **2**).

## Discussion

Intraocular tumors are uncommon and the types of intraocular tumors that occurred in the pediatric age group and the adult age group are different, which can often clinically lead to a misdiagnosis. In our selected cases, PTSU combined with MVRE was used as a primary globe-preserving treatment to prevent irradiation or enucleation and to conserve useful visual acuity. Some authors have reported the experience and outcome with local resection of intraocular tumors in the Caucasian population (3–12). However, most of the reported series (2–12) have not subcategorized patients with intraocular tumors based on the pediatric and adult age groups; particularly, the effectiveness of local resection in Asian population is still unknown.

PTSU combined with MVRE is successful for globe salvage, and is designed to remove tumor lesions, preserve vision and maintain a cosmetically normal eye. However, it is a surgically difficult procedure that can sometimes lead to adverse effects in the immediate and late postoperative period. Shields et al. (4) reported the postoperative course of 95 patients treated with PLSU for ciliary body and choroidal tumors. In their series, the most common intraoperative side effect was vitreous hemorrhage (83%) and subretinal or intraretinal hemorrhage (35%). Late postoperative side effects included cataract development in 34% and retinal detachment requiring surgery in 17%. A study by Naumann et al. (13) included 68 patients with iris and ciliary body tumors treated with en bloc excision. The main intraoperative issue in their study was vitreous hemorrhage in 35%, and the main postoperative side effect was cataract development in 32% patients. In our study, the most common immediate postoperative adverse side effect was vitreous hemorrhage (21%) and the most common postoperative adverse effect was PVR (18.3%). These side effects were often mild and had minor effects on the patients.

In literature concerning adult Caucasian patients regarding UM resection, globe-salvage has been achieved in 71% to 81% patients and final visual acuity of ≥20/40 is achieved in 50% to 53% patients (3, 9, 14). Ramasubramanian (5) reported 19 pediatric UM resections, with globe salvage in 76% patients and a final visual acuity of ≥20/40 in 64% patients. In our study on pediatric UM resection, globe salvage was reported in 80% patients, and final BCVA of ≥20/40 was achieved in 3.2% patients. In our study on adult UM resection, globe salvage was reported in 91.8% patients, and a final BCVA of ≥20/40 was achieved in 7.3% patients. The relatively low results for BCVA were associated with vitrectomy and a long follow-up duration.

In the present study, melanoma was the most common diagnosis (63.0%) in adult patients, followed by melanocytoma (7.3%), RPE adenoma (6.4%), and ciliary body adenoma of nonpigmented epithelium (5.7%). Melanoma was primarily located in the choroid (58.7%) and ciliary body (32.2%). Collaborative Ocular Melanoma Study (COMS) disclosed that melanoma-related mortality at 10 years was 17% to 18% for medium melanoma, and the patients underwent I-125 plaque brachytherapy, and 40% to 45% for large melanoma, wherein the patients underwent pre-enucleation radiation (15–18). In our previous study, we found the 5, 10-year UM metastasis rate and metastasis-related death (95% CI) in 1151 Asian UM patients were 15.5% (12.3%-19.5%), 24.5% (17.6% -33.6%); and 7.5% (5.3%-10.7%), 11.9% (8.1%-17.2%), respectively. Of these, 929 patients underwent the management of I-125 plaque brachytherapy (19). In this study, there were 213 medium-sized UM patients who underwent local resection, Kaplan-Merier analysis revealed that 5, 10-year metastasis rates and metastasis-related death rates (95% CI) in 213 UM patients were 3.2% (1.4%-7.0%), 6.9% (3.8%-12.3%); and 3.5% (1.6%-7.6%), 7.6% (4.2%-13.5%), respectively. The probable low rate of metastasis in this study maybe because of patient selection bias. Multivariate analysis revealed that the pathological type had no significant factors having different relative risks of metastasis and metastasis-related death in patients with UMs, the results were inconsistent with previous studies in which found that the presence of epithelioid tumor cells was one of the predictors (20). Considering the Racial differences and patient selection bias further studies were needed to evaluate its influence on UM onset among the Asian population. Iris tumors are less likely to metastasize and smaller tumors mainly located in the ciliary body are more preferable to be treated with PTSU combined with MVRE. In addition, during the mean follow-up duration of 24.1 months, a low recurrence rate of 7.7% (16/208) was noted, requiring subsequently rescue therapy. There was no metastasis-related death at the end of the follow-up. Based on the above mentioned findings, local resection has excellent effective outcomes.

In not completely conformity with the previous studies (5, 21–23), several special clinical features were noted in a large proportion of our pediatric patients: melanoma rarely occurred in the pediatric age group (n = 5), and no cases of metastatic disease and death were observed. However, UM was reported to occur with a frequency of 1.1% among all UMs in Caucasian patients<20 years of age. Of these, 12% to 21% arise in the iris and 88% to 79% from the ciliary body and choroid (21, 22). In a study by Shields et al. (22) including 106 patients with UM aged <20 years old, the rate of metastasis was 4.7% and the rate of death from metastasis was 2.8%. They noted that melanoma in the pediatric age group of patients was more likely to be pigmented, with melanoma located in the iris, more remote from the foveola and optic disc, and with a smaller tumor basal diameter and thickness (22). The rate of metastasis was significantly lower in patients aged <20 years (22).

Melanocytoma was the most commonly diagnosed in our pediatric age group; this result was inconsistent with a previous study (5). In Ramasubramanian’s (5) pediatric series, the most common diagnosis was medulloepithelioma. Frank et al. (23) reported the clinical features and management of 10 melanocytoma of the ciliary body. In their series, 1 patient was a child and the other 9 patients were adults. No recurrences were reported among the 10 patients who underwent management by iridocyclectomy. Melanocytomas of the ciliary body, although having the benign nature, have a propensity for invasion of the chamber angle structures and a tendency to recur. In this series, there were 16 pediatrics and 24 adult patients with melanocytoma; the mean largest tumor basal diameter was 11 mm vs 7 mm, and the mean tumor thickness was 4 mm vs 3 mm, respectively. Furthermore, we noted the different clinical features of melanocytoma between pediatrics and adults. Melanocytoma in pediatric patients was often with a larger tumor basal diameter and thickness, and more likely to be accompanied with hyphema. Further, 12 of the 16 pediatric patients (75%) showed local extension to the anterior chamber angle structures and intrascleral limbal plexuses. Of the 24 adult patients, 10 (41.7%) and 9 (37.5%) showed elevated preoperative IOP and optic disc atrophy, respectively. Moreover, 22 (91.7%) patients had remarkable pigmentation with dispersed fine dark spots distributed on the iris at presentation. Local extension or invasion of the chamber angle of the tumor was only observed in 2 (13%) patients. Pathological findings reported that mitosis was usually absent. Invasion of the chamber angle structures was observed histologically in 9 patients from the pediatric age group. Extensive necrosis was seen in 9 of 10 tumors, and malignant changes were noted in another. In contrast, these pathological features were not noted in the adult group.

In addition, although a melanocytoma of the ciliary body can usually be distinguished by histopathological criteria, clinically it may be difficult to distinguish from malignant melanoma before surgery. Like melanocytomas, ciliary body melanomas are usually slow-growing tumors (21, 22). Accordingly, we also do not rely on tumor growth as a differentiating factor. For the above reasons, if technically feasible, we favor iridocyclectomy or PTSU combined with MVRE over observation of darkly pigmented tumors of the ciliary body. Limbal incision was performed in all patients, and during the follow-up period, there was no recurrence was observed.

Medulloepithelioma was a relatively common diagnosis (21.6%) in pediatric patients. Medulloepithelioma is a congenital tumor that usually arises from the ciliary body, and most are accompanied with intratumoral cysts. In Ramasubramanian’s (5) series of 4 medulloepithelioma, they found medulloepithelioma was the most commonly misdiagnosed tumor preoperatively. In Zimmerman’s series (24) of 56 intraocular medulloepitheliomas, 45 (80%) were treated initially with iridocyclectomy but subsequently required enucleation: 20% developed extraocular extension and 7% died of tumor-related causes. In Canning’s (25) series of 16 patients with medulloepithelioma, 4 patients were initially treated with resection but all eventually needed enucleation. In our study, there were 9 patients with histopathological diagnosis of medulloepithelioma and 1 out of 9 patients ultimately required enucleation (globe salvage rate, 88.9%). None of the patients in our study developed extraocular disease or metastasis. We found that rubeosis is a common manifestation presented with medulloepithelioma (89%); thus, it maybe another clinical characteristic of this rare tumor.

The selected criteria and treatment guidelines we follow are based on experience from our patient series and from previous reports. Considering the controversy of fine-needle aspiration biopsy, given that there is potential for dissemination of tumor cells (26), we do not routinely choose to perform this procedure for speculated malignant lesions, including the simulated neoplastic lesions. In our series, there were 3 cases of adult-onset well-differentiated retinoblastoma without metastasis or death postoperatively, and the gene testing has confirmed the diagnosis lately. Regardless, an optimal treatment should be interpreted with caution because of the rarity of adult retinoblastoma.

In summary, PTSU combined with MVRE is an acceptable treatment option for benign and malignant intraocular tumors and simulating lesions in the pediatric age group and adult group, allowing globe-salvaging with the possibility of maintaining useful vision and favorable survival of the patients. The adverse effect rates are similar between the pediatric and the adult populations and enucleation is eventually needed in approximately 7.7% of patients. Patients with iris and ciliary body UM show benefits *via* local resection, and enucleation is needed only in few cases. Surgical resection of an intraocular tumor in its entirety with careful assessment of pathological features and gene analysis may have more potential benefits to favorable long-term outcomes.

## Sypnosis

In this work, we described the outcomes of intraocular tumor resection by partial transscleral sclerouvectomy (PTSU) combined with micro-invasive vitrectomy and reconstruction of the eyeball (MVRE) in 366 Asian patients in 25 years. The adverse effect rates were similar between the pediatric and the adult populations and enucleation waseventually needed in approximately 7.7% of patients. These findings suggested that local resection has excellent effective outcomes in patients with intraocular tumors.

## Data Availability Statement

The original contributions presented in the study are included in the article/**Supplementary Material**. Further inquiries can be directed to the corresponding author.

## Ethics Statement

The studies involving human participants were reviewed and approved by Institutional Review Board of Beijing Tongren Hospital. Written informed consent to participate in this study was provided by the participants’ legal guardian/next of kin. Written informed consent was obtained from the individual(s), and minor(s)’ legal guardian/next of kin, for the publication of any potentially identifiable images or data included in this article.

## Author Contributions

WW: Examination of patient, interpretation of results, writing the manuscript. NZ: Interpretation of results and writing/reviewing of manuscript. XX: Interpretation of results and reviewing of manuscript. PW: Reviewing of manuscript. YL: Examination and treatment of patient. All authors read and approved the final manuscript.

## Funding

The National Natural Science Foundation of China (Nr. 81272981), the Beijing Natural Science Foundation (Nr. 7151003) provided financial support. 

## Conflict of Interest

The authors declare that the research was conducted in the absence of any commercial or financial relationships that could be construed as a potential conflict of interest.

## Publisher’s Note

All claims expressed in this article are solely those of the authors and do not necessarily represent those of their affiliated organizations, or those of the publisher, the editors and the reviewers. Any product that may be evaluated in this article, or claim that may be made by its manufacturer, is not guaranteed or endorsed by the publisher.
