# Supplementary material for: Surgical Resection of Intraocular Tumors (Partial Transscleral Sclerouvectomy Combined With Mircoinvasive Vitrectomy and Reconstruction of the Eyeball) in Asian Patients: Twenty-Five Years Results
Source: Front Oncol. 2022 Mar 15;12:768635. doi: 10.3389/fonc.2022.768635 (PMC8965069; doi:10.3389/fonc.2022.768635)
Supplement: Supplementary file 1 [file Table_1.docx]

**Supplementary Table1.** Univariable and Multivariable Factors Influencing Metastasis in 213 Patients with Uveal Melanoma after Performing PTSU and MVRE

|  | **No. (%)** | |  |  |
| --- | --- | --- | --- | --- |
| **Feature** | **Metastasis**  **(n=13)** | **No Metastasis**  **(n=200)** | **HR (95%CI)** | **P Value** |
| **Univariable Analysis** | - | - | - | - |
| Age, mean, y | 33.7 | 43.6 | 0.95 [0.92-0.99] | 0.026 |
| Sex, male vs female | 7 | 106 | 0.92 [0.31-2.74] | 0.88 |
| Tumor base, mean, mm | 9.02 | 9.59 | 0.96 [0.80-1.15] | 0.68 |
| Tumor thickness, mean, mm | 7.02 | 6.81 | 1.06 [0.86-1.30] | 0.59 |
| Color (brown vs yellow) | 11 | 187 | 3.01 [0.67-13.62] | 0.15 |
| Pathological type | - | - | - | - |
| Epithelial vs spindle | 2 | 20 | 2.73 [0.50,15.03] | 0.25 |
| Mixed vs spindle | 7 | 101 | 1.50 [0.44, 5.14] | 0.52 |
| **Multivariable Analysis** | - | - | - | - |
| Age, mean, y |  |  | 0.96 [0.92-1.00] | 0.079 |
| Sex, male vs female |  |  | 0.82 [0.25-2.69] | 0.74 |
| Tumor base |  |  | 0.92 [0.72-1.18] | 0.52 |
| Tumor thickness |  |  | 1.08 [0.83-1.40] | 0.58 |
| Color (brown vs yellow) |  |  | 2.40 [0.45-12.79] | 0.30 |
| Pathological type | - | - | - | - |
| Epithelial vs spindle |  |  | 2.10 [0.34-13.10] | 0.43 |
| Mixed vs spindle |  |  | 1.73 [0.48-6.23] | 0.40 |
