# Supplementary material for: Surgical Resection of Intraocular Tumors (Partial Transscleral Sclerouvectomy Combined With Mircoinvasive Vitrectomy and Reconstruction of the Eyeball) in Asian Patients: Twenty-Five Years Results
Source: Front Oncol. 2022 Mar 15;12:768635. doi: 10.3389/fonc.2022.768635 (PMC8965069; doi:10.3389/fonc.2022.768635)
Supplement: Supplementary file 2 [file Table_2.docx]

**Supplementary Table 2.** Univariable and Multivariable Factors Influencing Death in 213 Patients With Uveal Melanoma after Performing PTSU and MVR

|  | **No. (%)** | |  |  |
| --- | --- | --- | --- | --- |
| **Feature** | **Death due to Metastasis**  **(n=15)** | **No Death**  **(n=198)** | **HR (95%CI)** | **P Value** |
| **Univariable Analysis** | - | - | - | - |
| Age, mean, y | 34.47 | 43.64 | 0.96 [0.92-1.00] | 0.033 |
| Sex, male vs female | 8 | 105 | 0.92 [0.33-2.54] | 0.88 |
| Tumor base, mean, mm | 9.33 | 9.57 | 1.00 [0.85-1.18] | >0.90 |
| Tumor thickness, mean, mm | 7.52 | 6.77 | 1.13 [0.95-1.35] | 0.18 |
| Color (brown vs yellow) | 13 | 185 | 2.50 [0.56-11.10] | 0.23 |
| Pathological type | - | - | - | - |
| Epithelial vs spindle | 2 | 21 | 2.05 [0.39-1.071] | 0.39 |
| Mixed vs spindle | 8 | 101 | 1.37 [0.45-4.19] | 0.58 |
| **Multivariable Analysis** | - | - | - | - |
| Age, mean, y |  |  | 0.97 [0.93-1.00] | 0.085 |
| Sex, male vs female |  |  | 0.91 [0.30-2.74] | 0.86 |
| Tumor base |  |  | 0.92 [0.74-1.16] | 0.49 |
| Tumor thickness |  |  | 1.16 [0.93-1.46] | 0.19 |
| Color (brown vs yellow) |  |  | 1.85 [0.36-9.59] | 0.46 |
| Pathological type | - | - | - | - |
| Epithelial vs spindle |  |  | 1.53 [0.26-9.04] | 0.64 |
| Mixed vs spindle |  |  | 1.56 [0.48-5.04] | 0.46 |
